# From Physique to Feelings: Deciphering the Body–Jealousy Connection in Women's Responses to Feminine Vocal Cues

**DOI:** 10.1002/pchj.70072

**Published:** 2026-01-04

**Authors:** Cairang Guanque, Chenle Xu, Chuhan Ji, Xingbang Ren, Xue Lei, Chengyang Han

**Affiliations:** ^1^ Jing Hengyi School of Education Hangzhou Normal University Hangzhou China; ^2^ School of Management Zhejiang University of Finance and Economics Hangzhou China; ^3^ Zhejiang Philosophy and Social Science Laboratory for Research in Early Development and Childcare Hangzhou Normal University Hangzhou China

**Keywords:** body size, intrasexual competition, jealousy, pitch, sexual dimorphism

## Abstract

Jealousy typically emerges when individuals sense that their romantic relationships may be threatened by others who display characteristics indicative of high mate quality. Previous research has found that in contexts of intrasexual competition, feminine female voices indicate high mate value and elicit stronger jealousy responses from other women. However, studies on individual differences in jealousy sensitivity are limited. Body size is an important factor that influences women's mating behavior. In the current study, we investigated the effect of women's height, weight, and body mass index (BMI) on their jealousy sensitivity to other women's vocal femininity. Results showed that women perceived more feminine voices as more jealousy‐inducing, and this effect was modulated by body size. Taller women demonstrated heightened sensitivity to vocal changes in pitch and formants, while slimmer women and those with a lower BMI showed increased sensitivity to pitch variations in competitive scenarios. These findings indicate that body size significantly shapes individual differences in jealousy sensitivity during intrasexual competition. Our study supports the mate quality–jealousy hypothesis, highlighting how traits perceived as indicators of higher mate quality amplify jealousy responses. The current research extends the literature on vocal cues and attractiveness by demonstrating how these factors influence emotional reactions such as jealousy.

## 1 Introduction

Jealousy is a pivotal psychological mechanism that influences romantic relationships. Jealousy typically emerges when individuals perceive that their relationship may be threatened by rivals who display traits signaling high mate quality (e.g., high physical attractiveness) (Dijkstra and Buunk 1998, 2001, 2002). These perceived threats elicit jealousy responses, which function to safeguard the relationship from potential rivals (Buss et al. 2000). Furthermore, the intensity of the jealousy response is associated with the perceived mate quality of the competitor. This relationship is encapsulated in the mate quality–jealousy hypothesis, which posits that rivals perceived as possessing higher mate quality elicit stronger feelings of jealousy from same‐sex individuals (Lei et al. 2019). Specifically, rivals who exhibit traits signaling enhanced mate quality tend to provoke more intense jealousy responses (O'Connor and Feinberg 2012). Exaggerated sexually dimorphic characteristics are often perceived as attractive (Little et al. 2011). Consequently, pronounced sexually dimorphic traits enhance perceived mate quality. Therefore, the presence of heightened sexual dimorphism is likely to evoke greater jealousy among same‐sex individuals (Lei et al. 2019; O'Connor and Feinberg 2012).

Human voices exhibit significant sexual dimorphism, with women's voices generally characterized by higher fundamental frequencies (i.e., perceived as voice pitch) and higher formant frequencies compared to men's (Chen et al. 2022; Puts 2016). Research has demonstrated that the acoustic properties of a voice not only signal gender but also influence perceptions of attractiveness. Higher‐pitched voices are typically perceived as more feminine, suggesting a link with feminine facial characteristics and overall desirability in a potential partner (Feinberg et al. 2005; Smith et al. 2016), though this is not always consistent (Pisanski et al. 2018). These vocal traits profoundly influence perceived mate quality because they serve as cues to underlying biological condition. For instance, a woman's higher pitch may signal good health and reproductive success, factors that are crucial when evaluating potential mates (Atkinson et al. 2012; Vukovic et al. 2010). The association between vocal pitch and reproductive health likely stems from biological mechanisms, particularly estrogen levels (Feinberg 2008). Estrogen modulates voice pitch, with higher levels enhancing vocal femininity (see Lindholm et al. 1997; see also Zamponi et al. 2021 for an overview). Additionally, elevated estrogen levels within the normal biological range are associated with fertility and the likelihood of successful conception (Venners et al. 2006). Consequently, a higher‐pitched voice may function as a signal of both reproductive potential and mate quality, reflecting a complex interplay of biological and perceptual factors (Abitbol et al. 1999; Atkinson et al. 2012; Pipitone and Gallup 2008; but see Puts et al. 2013). Importantly, it is well established that higher‐pitched voices in women are perceived as not only more feminine but also more attractive by both men and women (e.g., Puts et al. 2011).

Vocal femininity has been found to be positively correlated with jealousy. Research indicates that feminine‐sounding voices tend to provoke heightened feelings of jealousy among heterosexual women when they envision situations in which their romantic partners are interacting with women who possess high‐pitched voices (O'Connor and Feinberg 2012; Puts et al. 2011). In addition, women appear to entertain more aggressive thoughts toward other women who exhibit higher‐pitched voices, particularly when primed with a long‐term mating motive (Zhang 2016). Recently, a cross‐cultural study suggested that women perceive higher‐pitched female voices as sounding more flirtatious toward men (Aung et al. 2024). While studies on women's voices and jealousy have mainly focused on pitch, limited research has investigated the relationship between formant frequencies and jealousy. Notably, one study reported that formant frequencies had a larger effect on attractiveness and flirtatiousness than fundamental frequency (F0) did (Puts et al. 2011). In the current study, we tested women's sensitivity to other women's vocal femininity (both F0 and formant frequencies) by examining their jealousy judgments.

Women's jealousy may also be influenced by other individual differences, such as body size. Body size has been shown to influence human social cognition and decision‐making (e.g., Watkins et al. 2010; Brewer and Riley 2009). Although women's height can influence their jealousy judgments, the results are mixed. Some studies reported that women's height was negatively associated with jealousy, while others found a non‐linear, quadratic association (Buunk et al. 2008, 2019; Pavela et al. 2014). A limitation of these studies is their reliance on self‐report questionnaires, which typically measure general, trait‐level jealousy rather than a direct response to a rival's cues. Our study moves beyond this by introducing a behavioral paradigm to assess cue‐specific jealousy sensitivity. Specifically, we operationalize *jealousy sensitivity* as the strength of the relationship between objective, continuous acoustic parameters of a rival's voice (i.e., F0 and format position (Pf), Puts et al. 2012) and a participant's jealousy ratings. A stronger association, indicated by a steeper slope in our statistical model, represents heightened sensitivity to these specific vocal cues of threat. Additionally, there is limited information available in the literature regarding how women's body weight influences their jealousy perception. Therefore, in the current study, we examined how women's height, body weight, and BMI influence their jealousy sensitivity.

While the link between a rival's traits and jealousy is established, the factors that shape individual differences in jealousy sensitivity remain poorly understood. We propose that a woman's own physique, which is a key component of her mate value, functions as a critical moderator. The theoretical rationale for this moderation extends beyond a simple main effect (i.e., that more attractive women are dispositionally more jealous). Instead, we argue from a functional, cost‐benefit perspective that jealousy operates as a calibrated threat‐detection system.

From this perspective, women with physical traits associated with higher mate value (e.g., taller stature, lower body mass) are more likely to secure and maintain relationships with high‐value partners (Buss and Shackelford 2008). These desirable partners, in turn, are subject to more frequent and intense mate poaching attempts from rivals (Krems et al. 2016). For a woman paired with a high‐value partner, the potential cost of failing to detect a formidable rival is substantial. Consequently, natural selection may have favored a psychological mechanism that is not merely hyper‐vigilant at all times, but is instead highly sensitive to gradations in a rival's quality. This heightened sensitivity would manifest as a stronger emotional response to incremental increases in a rival's threat cues, such as increasing vocal femininity.

Therefore, we did not hypothesize that women with certain physical characteristics would report higher jealousy overall, but rather that their jealousy ratings would be more strongly associated with the acoustic cues. Specifically, given that taller stature and slenderness are perceived as attractive in many contemporary societies (e.g., Buunk et al. 2019; Han et al. 2021, 2023), we proposed the following hypotheses:

*Women's jealousy ratings will be positively associated with the femininity of a rival's voice* (*as indexed by* *F0* *and Pf*).

*This positive association will be moderated by the participant's own body size. Specifically, the relationship between vocal femininity and jealousy will be stronger for* (*a*) *taller women and* (*b*) *women with lower weight and BMI*.

## 2 Methods

### 2.1 Participants

A total of 134 heterosexual women, aged between 18 and 40 years (*M* = 19.36, SD = 2.68), were recruited from a university campus to participate in the study. An a priori power analysis was conducted using G*Power (version 3.1; Faul et al. 2009) to determine the required sample size. The analysis, based on an *F*‐test for linear multiple regression (fixed model, *R* ^2^ increase), aimed to detect a small to medium effect size (Cohen *f* ^2^ = 0.1) for our key interaction terms. With an alpha level set at 0.05 and a desired power of 0.95, the analysis indicated that a minimum sample size of 132 participants was required. Thus, our final sample of 134 participants provided adequate statistical power for the main analyses. Individuals with a history of psychiatric disorders or those currently taking psychotropic medications were excluded. All participants reported normal hearing and normal or corrected‐to‐normal vision. Participants received 40 Chinese yuan per hour for their participation. The study was approved by the Ethics Committee of Hangzhou Normal University and was conducted in accordance with the Declaration of Helsinki.

### 2.2 Voice Stimuli

The stimuli consisted of 131 voice recordings obtained from young adult Chinese women (ages 18–35). Recordings were made using an Audio‐Technica AT‐4041 cardioid condenser microphone at a sampling rate of 44.1 kHz with 16‐bit amplitude quantization. The spoken word “Hi” was extracted from each recording to serve as the auditory stimulus. The sound pressure level (SPL) of all voice clips was normalized to 70 dB using the root‐mean‐square (RMS) method. Acoustic measurements were conducted using Praat (Boersma and Weenink 2013). Specifically, fundamental frequency (F0) was determined using Praat's autocorrelation algorithm with a search range of 100–600 Hz (Pisanski et al. 2014). F1–F4 were measured using the Burg linear predictive coding (LPC) algorithm in Praat, with the maximum formant set to 5500 Hz. Formants were initially overlaid on a spectrogram, and the formant numbers were manually adjusted for the best visual alignment between predicted and observed formants. These methods and settings are recommended by the Praat manual (Boersma and Weenink 2013; see also Pisanski et al. 2014). Formant position (Pf) was calculated as described by Puts et al. (2012).

### 2.3 Voice Judgment Task

In the voice judgment task, participants were given the following instructions (translated from Chinese): “You will hear women's voices. Please rate how jealous you would be (from 1= *low* to 7 = *high*) if she were flirting with your romantic partner. If you are not currently in a romantic relationship, please imagine that you are” (following O'Connor and Feinberg 2012). The task consisted of 131 trials. In each trial, a single voice stimulus was presented, after which the participant provided their jealousy rating. This procedure was repeated for all 131 unique voice stimuli, presented in a randomized order for each participant, yielding 131 within‐subject observations per participant. All voices were presented via headphones at a constant volume.

### 2.4 Procedure

Prior to the experiment, all participants provided written informed consent. The jealousy judgment task was programmed using PsychoPy (Peirce et al. 2019) and was administered on computers in quiet, individual testing rooms. The entire experimental session lasted approximately 10 min. Following the voice judgment task, participants' height (range: 140–180 cm, *M* = 164.28, SD = 7.26) and weight (range: 40–74 kg, *M* = 52.67, SD = 7.89) were measured using a stadiometer and a scale, respectively. BMI was subsequently calculated (range: 14.87–27.55 kg/m^2^, *M* = 19.56, SD = 2.97).

### 2.5 Statistical Analysis

Linear mixed models were utilized to examine how body size influences the association between jealousy judgments and sexually dimorphic vocal traits. Analyses were performed using R version 4.3.2 (R Core Team 2023) and the lmerTest package version 3.10 (Kuznetsova et al. 2017). Prior to analysis, participants' height, weight, and BMI were standardized (*z*‐scored); the vocal parameter F0 was also standardized, while Pf was not standardized, as it is inherently derived from the average of the *z*‐scores of F1–F4 (Puts et al. 2012).

We analyzed weight, BMI, and height independently, resulting in three models. Each model had the same dependent variable: jealousy ratings ranging from 1 (low) to 7 (high). In the weight model, the fixed‐effect factors included: weight (*z*‐scored), F0 (*z*‐scored), Pf, and all two‐way interactions among them. The BMI model was identical to the weight model, with BMI substituted for weight. Similarly, the height model followed the same structure, replacing weight with height. Subject ID and voice ID were included in the model as random‐effect factors, in accordance with Barr et al. (2013) and Barr (2013).

## 3 Results

### 3.1 Height

**FIGURE 1:**
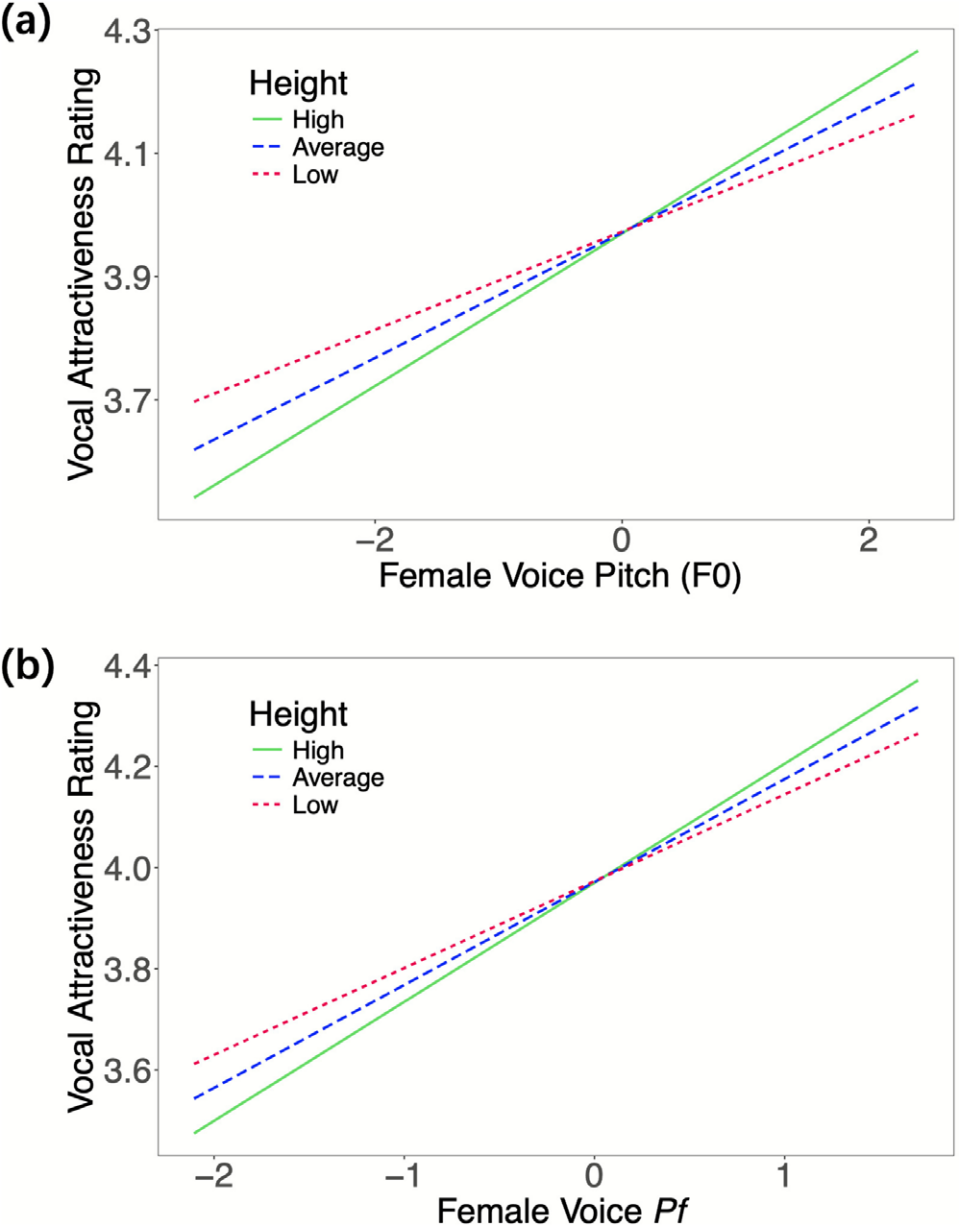
(a) The effect of women's height on the relationship between F0 and jealousy ratings. (b) The effect of women's height on the relationship between Pf and jealousy ratings. The value of the slope was higher in taller women than it was in shorter women, indicating that taller women were more sensitive to F0 changes and Pf changes than shorter women were in jealousy judgments. High height = 2 SD above the mean, low height = 2 SD below the mean.

The results of the height model are presented in Table 1. There was a significant interaction between F0 and height (*b* = 0.02, SE = 0.01, *t*(133.32) = 2.17, *p* = 0.032), and Pf and height (*b* = 0.03, SE = 0.01, *t*(129.92) = 2.16, *p* = 0.033). This indicates that women's height moderates the association between vocal cues (F0, Pf) and jealousy perception. Specifically, the positive association between F0 and jealousy ratings was weaker for shorter women compared to taller women (see Figure 1a). Similarly, the positive association between Pf and jealousy ratings was weaker for shorter women compared to taller women (see Figure 1b). Additionally, the main effect of vocal femininity was replicated in this model, with both F0 and Pf showing a positive association with jealousy ratings (see Table 1).

**TABLE 1:** The results of height model.

Height model						
	*b*	SE	df	*t*	*p*	
Intercept	3.97	0.06	230.21	63.44	< 0.001	***
Height	0.00	0.05	135.41	−0.03	0.976	
F0	0.10	0.04	130.55	2.67	0.008	**
Pf	0.20	0.05	130.55	3.75	< 0.001	***
Height × F0	0.02	0.01	133.32	2.17	0.032	*
Height × Pf	0.03	0.01	129.92	2.16	0.033	*
F0 × Pf	−0.05	0.06	142.46	−0.79	0.433	

*Note:* *b* = unstandardized coefficient; SE = standard error; df = degrees of freedom. *p < 0.05; **p < 0.01; ***p < 0.001.

### 3.2 Weight

**FIGURE 2:**
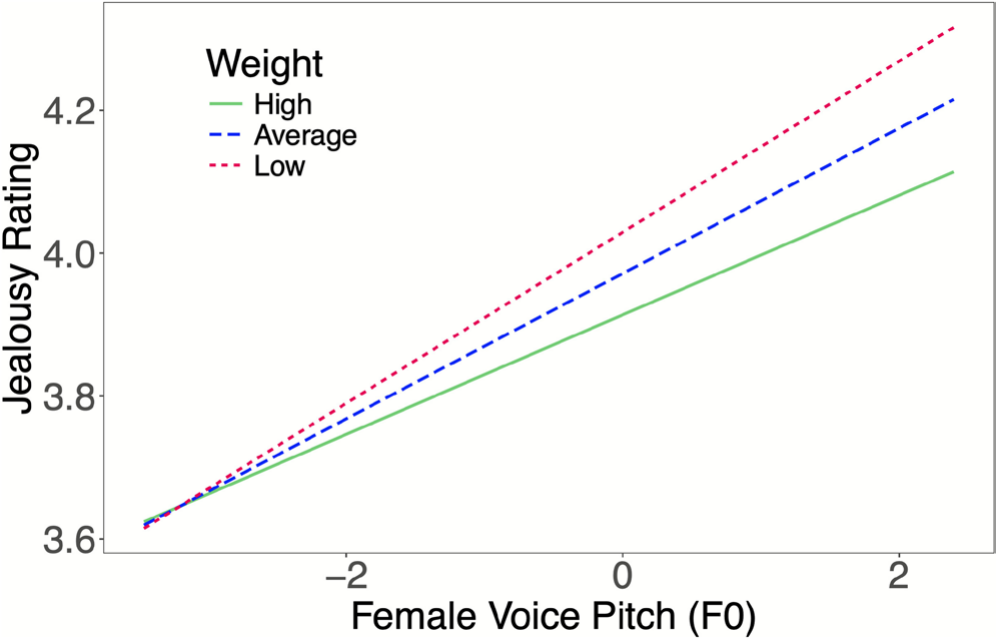
The effect of women's weight on the relationship between F0 and jealousy ratings. The value of the slope was lower in high weight women than it was in low weight women, indicating that low weight women were more sensitive to F0 changes than high weight women in jealousy judgments. High weight = 2 SD above the mean, low weight = 2 SD below the mean.

The results of the weight model are presented in Table 2. There was a marginally significant interaction between F0 and weight (*b* = −0.02, SE = 0.01, *t*(134.60) = −1.94, *p* = 0.055), indicating that women's body weight moderated the association between F0 and jealousy perception. Specifically, the positive association between F0 and jealousy ratings was weaker for women with higher weight compared to women with lower weight (see Figure 2). This result suggests that jealousy sensitivity is negatively associated with weight; specifically, slimmer women are more sensitive to pitch variations in their jealousy judgments. Moreover, both F0 and Pf were positively associated with jealousy ratings (see Table 2), demonstrating that female voices with high F0 and high Pf (i.e., feminine voice) elicited higher jealousy ratings than voices with low F0 and low Pf.

**TABLE 2:** The results of weight model.

Weight model						
	*b*	SE	df	*t*	*p*	
Intercept	3.97	0.06	230.67	63.64	< 0.001	***
Weight	−0.06	0.05	133.89	−1.12	0.264	
F0	0.10	0.04	130.53	2.67	0.008	**
Pf	0.20	0.05	130.53	3.75	< 0.001	***
Weight × F0	−0.02	0.01	134.60	−1.94	0.05	+
Weight × Pf	0.01	0.01	131.30	0.44	0.663	
F0 × Pf	−0.05	0.06	142.05	−0.79	0.432	

*Note:* *b* = unstandardized coefficient; SE = standard error; df = degrees of freedom. + *p* < 0.10; **p < 0.01; ***p < 0.001.

### 3.3 BMI

**FIGURE 3:**
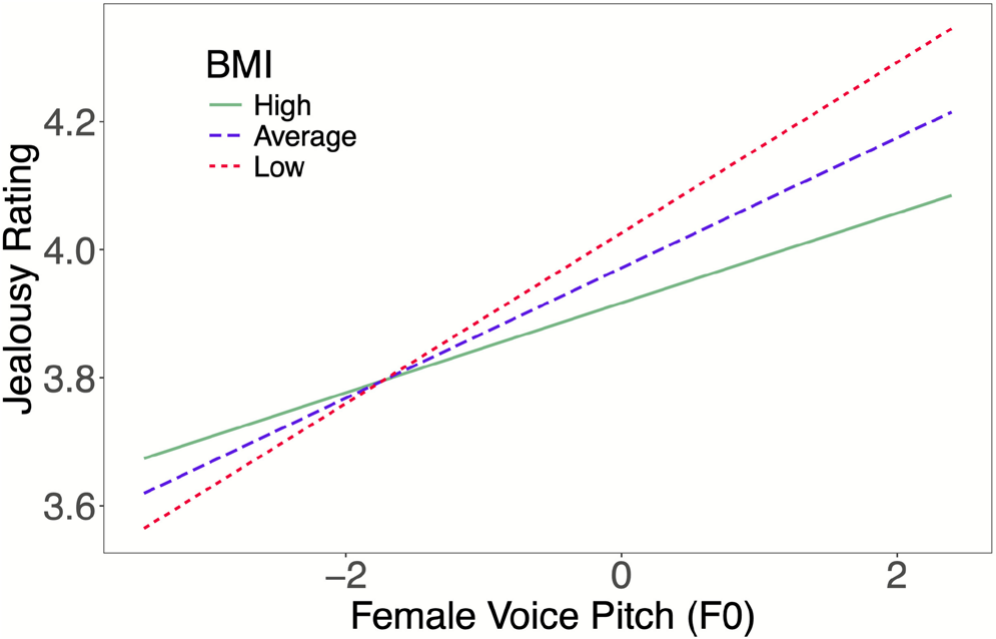
The effect of women's BMI on the relationship between F0 and jealousy ratings. The value of the slope on both figures was lower in high BMI women than it was in low BMI women, indicating that women with low BMI were more sensitive to F0 changes than women with high BMI were in jealousy judgments. High BMI = 2 SD above the mean, low BMI = 2 SD below the mean.

The results of the BMI model are presented in Table 3. There was a significant interaction between F0 and BMI (*b* = −0.03, SE = 0.01, *t*(134.20) = −3.13, *p* = 0.002), indicating that women's BMI moderated the association between F0 and jealousy perception. Specifically, the positive association between F0 and jealousy ratings was weaker for women with a higher BMI compared to those with a lower BMI (see Figure 3). This result indicates that jealousy sensitivity is negatively associated with participant BMI; specifically, women with a lower BMI are more sensitive to pitch variations in their jealousy judgments. Additionally, the main effect of vocal femininity was replicated in this model, with both F0 and Pf showing a positive association with jealousy ratings (see Table 3).

**TABLE 3:** The results of the BMI model.

BMI model						
	*b*	SE	df	*t*	*p*	
Intercept	3.97	0.06	230.64	63.62	< 0.001	***
BMI	−0.05	0.05	135.28	−1.06	0.292	
F0	0.10	0.04	130.55	2.67	0.008	**
Pf	0.20	0.05	130.55	3.75	< 0.001	***
BMI × F0	−0.03	0.01	134.20	−3.13	0.002	**
BMI × Pf	−0.01	0.01	130.88	−1.00	0.318	
F0 × Pf	−0.05	0.06	142.33	−0.79	0.433	

*Note:* *b* = unstandardized coefficient; SE = standard error; df = degrees of freedom. **p < 0.01; ***p < 0.001.

## 4 Discussion

This study investigated how a woman's own body size (i.e., height, weight, and BMI) moderates her jealousy sensitivity to a potential rival's vocal cues. Our results yielded two key findings. First, consistent with previous research (e.g., O'Connor and Feinberg 2012; Puts et al. 2011), we found that women perceived voices as more jealousy‐inducing when the voices were more acoustically feminine (i.e., higher in F0 and Pf). Second, and more importantly, we demonstrated for the first time that this association is moderated by the participants' own physique. Specifically, taller women exhibited heightened sensitivity to changes in both pitch (F0) and formants (Pf), while slimmer women (i.e., those with lower weight and BMI) showed heightened sensitivity specifically to pitch variations. These findings provide novel evidence that an individual's own physical traits play a significant role in calibrating jealousy sensitivity during intrasexual competition.

The positive association we found between vocal femininity (both F0 and Pf) and jealousy ratings is consistent with prior research (O'Connor and Feinberg 2012; Puts et al. 2011). However, our study explored further by investigating how individual differences in body size moderate these relationships. Taller, slimmer, and lower BMI women exhibited heightened sensitivity to feminine vocal cues, suggesting that body size is an important factor in shaping women's perceptions of potential rivals' mate value and their subsequent jealousy responses.

### 4.1 Height and Jealousy Sensitivity

The positive association we found between women's height and their sensitivity to vocal femininity can be explained by the sociocultural perception of height as an attractive and desirable trait. Taller stature (though typically within a normative range) is often viewed as a symbol of beauty and high mate value in many societies (e.g., Courtiol et al. 2010; Cawley et al. 2006). Taller women are often more physically conspicuous, causing them to be perceived as formidable competitors in the mating market (Smits and Monden 2012). This heightened visibility and perceived attractiveness may expose taller women to increased intrasexual aggression from female rivals (Zhang 2016). Consequently, they may have developed heightened vigilance toward cues that signal potential threats from other women, such as vocal femininity. Thus, taller women may be more attuned to feminine vocal characteristics because these cues signal a significant threat to their romantic relationships and access to high‐quality mates (O'Connor and Feinberg 2012).

Additionally, due to assortative mating, taller women tend to pair with taller partners, who are generally perceived as more attractive (Salska et al. 2008; Stulp and Barrett 2016). Partnering with such desirable mates may further amplify women's jealousy sensitivity, functioning as a mechanism for effective mate guarding. Because high‐value partners are frequently targeted by rivals, taller women may exhibit a heightened drive to safeguard their relationships from potential competitors.

The increased sensitivity to vocal cues of femininity may represent an adaptive strategy enabling women to safeguard access to high‐quality mates (Buss 2002; O'Connor and Feinberg 2012). By detecting signals of intrasexual competition more acutely, women can optimize their mate‐guarding behaviors and increase the likelihood of relationship retention. In summary, the positive relationship between women's height and their jealousy sensitivity to vocal femininity underscores how the sociocultural valuation of tall stature as a desirable trait amplifies tall women's vigilance toward potential rivals and their drive to protect their romantic partnerships.

However, previous research on the association between women's height and jealousy has yielded mixed results. While some studies have reported a negative relationship, others have found a more complex, quadratic pattern (Buunk et al. 2008, 2019; Pavela et al. 2014). In the current study, we observed that height was positively associated with jealousy sensitivity. These discrepancies may be partly explained by differences in methodological approaches. Previous studies relied on self‐report questionnaires to measure women's general jealousy intensity (Buunk et al. 2008, 2019; Pavela et al. 2014). In contrast, our study utilized an objective measure of jealousy sensitivity to specific vocal cues, rather than focusing solely on the association between height and trait jealousy. Our methodology offers a novel approach to understanding how height influences women's jealousy responses in the context of intrasexual competition.

### 4.2 Weight, BMI and Jealousy Sensitivity

Our results suggest that women's weight and BMI play a significant role in shaping their jealousy sensitivity to vocal cues of femininity. Specifically, lower weight and lower BMI were associated with heightened sensitivity to pitch changes in jealousy‐inducing scenarios. This pattern aligns with the sociocultural perception of slenderness (low weight and BMI) as a desirable and attractive trait in women (Swami and Tovée 2005; Han et al. 2021). Just as tall stature is viewed as a sign of high mate value, a slender physique is strongly associated with high attractiveness (Swami and Tovée 2005; Han et al. 2021). Consequently, slimmer women are perceived as more attractive and may face increased threats and aggression from female rivals (Zhang 2016), as they are viewed as more formidable competitors in the mating market. Over time, this heightened exposure to intrasexual aggression may lead them to become more vigilant and sensitive to signals indicating a threat from other women, such as vocal cues of femininity (O'Connor and Feinberg 2012).

Furthermore, the relative physical disadvantage associated with lower body weight may also contribute to these women's heightened sensitivity to potential threats. Compared to their heavier counterparts, slimmer women typically possess lower physical formidability, making them more vulnerable in direct physical confrontations. The critical importance of body mass in determining physical dominance is well illustrated by the strict weight class divisions maintained in combat sports (e.g., boxing). Consequently, due to this potential physical vulnerability, slimmer women may have evolved heightened sensitivity to social threat cues—such as vocal femininity—as a compensatory mechanism. This vigilance allows them to safeguard their romantic relationships and access to high‐quality mates while minimizing the need for direct physical aggression (Buss 2002).

Overall, the findings regarding weight and BMI mirror the patterns observed for height, indicating that women's body size plays a crucial role in shaping their sensitivity to vocal cues of femininity in jealousy judgments. Women with lower weight and BMI may face greater aggression in intrasexual competition and, therefore, may have developed heightened sensitivity to potential threats from rivals. Moreover, previous studies have primarily focused on the relationship between height and jealousy (Buunk et al. 2008, 2019; Pavela et al. 2014), with limited exploration of the role of weight or BMI. Our study extends the literature by examining how both weight and BMI influence women's jealousy sensitivity, providing a comprehensive understanding of the complex interplay between body size and jealousy.

### 4.3 Theoretical Implications

Our results lend support to the mate quality‐jealousy hypothesis, which posits that rivals perceived as possessing higher mate quality are more likely to elicit stronger jealousy responses (Lei et al. 2019). The positive associations we observed between women's taller stature, lower weight, and lower BMI—and their heightened sensitivity to vocal femininity cues—suggest that these physical characteristics are indeed interpreted as signals of high mate quality. Consequently, women possessing these traits may be viewed as more formidable competitors. In turn, they may have developed heightened vigilance toward threats from potential rivals, as attractive women often face more intense intrasexual competition (Zhang 2016).

Our findings align with the evolutionary perspective emphasizing the critical role of physical attributes in mate selection and intrasexual competition. Taller stature, lower weight, and lower BMI are frequently associated with youth, health, and fertility—all of which are desirable characteristics in a mate (Han et al. 2021; Salska et al. 2008; Swami and Tovée 2005). We suggest that for women possessing these traits, heightened sensitivity to vocal cues of femininity serves as an adaptive mate‐guarding strategy to protect access to high‐quality partners. Specifically, this heightened jealousy sensitivity may function as an evolved psychological mechanism, enabling taller and slimmer women to effectively navigate the landscape of intrasexual competition and retain their advantageous position in the mating market (Buss et al. 2000; Buss 2002).

Additionally, the patterns we observed may also reflect the internalization of sociocultural beauty norms that idealize certain body types, such as tall stature and slimness (Han et al. 2021; Salska et al. 2008; Swami and Tovée 2005). These societal standards can shape women's self‐perceptions and their evaluation of potential rivals, leading them to be more attuned to vocal cues that signal the presence of more desirable mates.

### 4.4 Limitations and Future Directions

Our study deliberately focused on isolated vocal cues to disentangle the specific contribution of vocal femininity to jealousy, a methodological approach that is essential for establishing a clear causal link without the confounding influence of visual information (e.g., facial attractiveness, body language). While we acknowledge that many real‐world social encounters are multi‐modal, there are numerous ecologically valid scenarios where auditory cues are primary or presented in isolation. For instance, jealousy can be triggered by overhearing a partner's phone conversation, interacting via voice notes on dating apps before meeting, or engaging in online gaming where vocal cues are dissociated from physical appearance. Therefore, understanding the impact of vocal cues alone has significant practical relevance. This focused investigation provides a critical baseline, laying the groundwork for future multi‐modal research designed to explore the complex and potentially interactive effects of visual and auditory signals on intrasexual competition.

While this study provides novel insights, several limitations should be acknowledged, which also point to valuable directions for future research. First, we did not collect data on participants' menstrual cycle phase. Given that hormonal fluctuations across the menstrual cycle are known to influence women's mating‐related perceptions, attractiveness judgments, and competitive behaviors (e.g., Gildersleeve et al. 2014; Jones et al. 2008), future studies could examine how jealousy sensitivity to vocal cues varies as a function of hormonal status. Second, our use of weight and BMI as proxies for physique does not differentiate between muscle mass and adipose tissue. Future investigations could employ more direct measures of body composition, such as waist‐to‐hip ratio (WHR), which is a strong signal of female health, fertility, and attractiveness (e.g., Singh 1993), to provide a more nuanced understanding of this relationship.

Finally, our study focused specifically on physical characteristics as moderators, yet jealousy is a multi‐faceted emotion influenced by a wide array of factors. A more comprehensive model would need to incorporate other individual differences. For example, personality traits such as neuroticism or self‐esteem (e.g., Buunk 1997), and relationship‐specific factors like relationship satisfaction or investment (e.g., Rydell and Bringle 2007), are all likely to shape jealousy responses. Future research integrating these psychological and relational variables with physical traits would provide a much richer understanding of the antecedents of jealousy sensitivity in the context of intrasexual competition.

### 4.5 Conclusion

Overall, our findings contribute to a more nuanced understanding of the complex interplay among women's body size, vocal femininity, and jealousy sensitivity. By incorporating both height and weight/BMI measures, as well as objective behavioral assessments of jealousy, we offer a more comprehensive and ecologically valid approach compared to previous research relying on self‐report measures. These insights have the potential to inform theoretical models of intrasexual competition, as well as practical applications in relationship counseling and social dynamics.

## Funding

Chengyang Han was supported by the Natural Science Foundation of Zhejiang Province (LQ23C090004) and the National Natural Science Foundation of China (32300902). The funding sources had no further role in the study design, data collection, analysis, interpretation, and decision to submit this manuscript for publication.

## Conflicts of Interest

The authors declare no conflicts of interest.

## Data Availability

The data that support the findings of this study are available from the corresponding author upon reasonable request.
